# Urea, Its Tests and Origin

**Published:** 1851-10

**Authors:** 


*Urea, its Tests and Origin.* By Prof. Lehmann.—Urea may generally be very easily recognised by its properties, especially by its behavior towards nitric and oxalic acids; but when we have to discover very minute quantities of this substance in albuminous fluids, it is often very difficult to determine its presence with scientific precision. It is in alcoholic extracts that we must always seek for urea; but before we proceed to search for it, there are several precautionary measures to be adopted, the neglect of which would render our attempt to discover it futile. In the first place, in reference to the presence of albuminous substances, if we wish to discover small quantities of urea in albuminous fluids, we must not be satisfied with the removal of the albumen by simple boiling; since, by the coagulation of the albumen, the fluid becomes more alkaline, and might, during evaporation, induce a decomposition of the urea; moreover, all albuminous matter is not precipitated by boiling, but a portion remains dissolved by the alkali, and is taken up in the alcoholic extract. On evaporation, this albumen undergoes a change, which probably coöperates with the alkali in inducing the decomposition of the urea. This may explain how it was that Marchand could only recover 0.2 of a gramme of urea from a mixture of 200 grammes of serum and 1 gramme of urea. Hence, before boiling the albuminous fluid, we must add a few drops of acetic acid, so as to give it a slightly acid reaction, whereby not only is the alkalescence of the fluid prevented, but a much more perfect separation of the coagulable matters is effected. If the residue of the fluid from which the coagulated matters have been filtered be extracted with cold alcohol, and the solution rapidly evaporated, so as to cause the chloride of sodium (taken up by the cold alcohol) to separate as much as possible in crystals, on then bringing a drop of the mother-liquid in contact with nitric acid under the microscope, we shall observe the commencement of the formation of the rhombic octohedra, and the hexagonal tablets, in which, if the investigation is to be unquestionable, the acute angles ($=82°$) must be always measured. After determination of the nitrate, we may also obtain the oxalate, and submit it to microscopic examination. A good crystallometric determination yields, however, the same certainty as an elementary analysis, which, in these cases, would never or extremely seldom be possible. The investigations of Marchand have thrown much light upon this subject [the seat of the actual formation of urea]. This accurate observer could only recover 0.2 of a gramme of urea from 200 grammes of serum, to which 1 gramme of urea had been added. He shows that, even if the urea were only separated from the blood at the end of each successive hour, it could not have accumulated in such quantity as to have been discoverable by the present mode of investigation. The following consideration will give us an idea of the small quantity of urea which, according to Marchand's hypothesis, at the most can accumulate in the blood in one hour:—From the experiments of Ed. Weber, which I have in part confirmed, we may assume that there are, in an adult man, at most 6 or 7 kilogrammes [16 to 19 pounds] of circulating blood. Now, if, in twenty-four hours, 30 grammes of urea are discharged, at most only 1.25 grammes could accumulate in one hour in the whole mass of the blood; so that only 0.021 could be contained in it. This minute quantity can, however, as we have already shown, only be detected in operating on very large masses of blood and by the aid of the microscope. Hence it is easy to understand why, during my experiments with an animal diet, while the urine was loaded with urea, none of this substance could be discovered in the blood.

If it be now established that the urea is not primarily formed in the kidneys, the question still remains to be answered, whether it is produced in the circulating blood or in the individual living organs, (as, for instance, the muscles,) and from what materials it is principally formed. In the present state of our knowledge, we may answer, that the urea is formed in the blood, and that it is produced from materials that have become effete, the detritus of tissues, as well as from unserviceable and superfluous nitrogenous substances in the blood. No animal tissue presents such vital activity, is so much used, and is so rapidly worn out, as muscular tissue; it is in this tissue that the metamorphosis of matter proceeds most rapidly and abundantly; and yet, in the large quantities of muscular fluid on which Liebig worked, he could detect no trace of urea, although he found substances from which he could produce urea artificially. We must therefore assume that these substances, as creatine and probably inosinic acid, are decomposed in the blood, by the action of the alkalies and of free oxygen, into urea and other matters to be excreted. Moreover, my experiments, showing that the superfluous nitrogenous food which enters the blood, and the fact that caffeine, glycine (Horsford), uric acid, and alloxantin (Wohler and Frerichs), soon after they have been taken, perceptibly increase the amount of urea in the urine, support the view that urea is formed in the blood. It is impossible to suppose that this nitrogenous food is first converted into tissue, and subsequently into urea, &c.; for we cannot think that a process occurs here, analogous to that exhibited by the percussion-apparatus of the physicists, where a certain number of parts effecting a percussion give rise to the repulsion of an equal number of parts. Hence the conversion of this matter can occur in no other place than in the circulating blood, and therefore it is here that the urea must be formed.

That the urea is formed from nitrogenous matters could not be doubted, even if it did not contain nitrogen (and that in so large a quantity); for it is especially after the use of highly nitrogenous food, that we find an augmentation of its quantity in the urine. If, however, we should further inquire,—from what substances is it produced, and what tissues principally contribute to its formation?—we could not, in the present state of our knowledge, give any satisfactory answer to this question. All that we know is, that urea is a very general product of the decomposition of nitrogenous matters, both naturally within the animal body, and artificially in the laboratory of the chemist. We have already said enough to show, that urea is so common a product of the decomposition of nitrogenous bodies, that we could hardly any longer enumerate it among true organic substances, if we tried to establish a distinction between organic and inorganic matter. Moreover, when we treat of uric acid, we shall show, that, in all probability, a great part of the urea separated by the kidneys from the blood is the product of the decomposition of that acid.—*Med. Chir. Rev.*

